# Optimisation and field validation of odour-baited traps for surveillance of *Aedes aegypti* adults in Paramaribo, Suriname

**DOI:** 10.1186/s13071-020-4001-y

**Published:** 2020-03-06

**Authors:** Tessa M. Visser, Marieke P. de Cock, Hélène Hiwat, Merril Wongsokarijo, Niels O. Verhulst, Constantianus J. M. Koenraadt

**Affiliations:** 1 Laboratory of Entomology, Department of Plant Sciences, Wageningen University and Research, Wageningen, The Netherlands; 2 Malaria Programme, Ministry of Health, Paramaribo, Suriname; 3 Bureau of Public Health, Ministry of Health, Paramaribo, Suriname; 4 National Centre for Vector Entomology, Institute of Parasitology, Vetsuisse Faculty, University of Zürich, Zurich, Switzerland

**Keywords:** *Aedes aegypti*, Carbon dioxide, Host-seeking, Trapping, Odour-baited traps, Odour-blends

## Abstract

**Background:**

Emerging arboviral diseases like Zika, dengue and chikungunya that are transmitted by *Aedes aegypti* mosquitoes, are increasingly threatening human health. Blends of human-like synthetic chemical attractants can be used to attract host-seeking mosquitoes. The aim of this study was to test new combinations of traps and odour baits in the laboratory, followed by testing the best candidates in the field to improve *Ae. aegypti* monitoring and surveillance.

**Methods:**

First, the BG-Suna trap was evaluated for capturing laboratory-reared *Ae. aegypti* by testing normal and inverted positions in screen cage tests. Secondly, the attractiveness of the MB5 blend, CO_2_, and their combination was tested. Thirdly, we tested the attractiveness of different trap types (BG-Suna, BG-Sentinel, MM-X and CDC light trap). Finally, we confirmed laboratory results in the field in Paramaribo, Suriname, using the MB5 and BG-Lure odour blends, CO_2_ and the BG-Sentinel and BG-Bowl trap using a Latin Square design.

**Results:**

The MB5 blend in combination with CO_2_ outperformed traps baited only with CO_2_ or MB5 in screen cage tests (*P* < 0.0001). The BG-Sentinel trap performed equally well as the inverted BG-Suna and was taken to the field (*P* = 0.729). In the field, we captured *Ae. aegypti*, *Cx. quinquefasciatus* and *Cx. nigripalpus*. We confirmed the laboratory results and found that the combination of the MB5 blend and CO_2_ almost doubled *Ae. aegypti* female captures (*P* = 0.004) and more than doubled *Culex* spp. female captures (*P* = 0.005) compared to using only CO_2_. Interestingly, the MB5 blend outperformed the commercially available BG-Lure, in the BG-Sentinel (*P* < 0.001). The BG-Bowl also attracted *Ae. aegypti* when baited with the MB5 blend in similar numbers as the BG-Sentinel baited with the MB5 (*P* = 0.362).

**Conclusions:**

Our study demonstrated that the BG-Sentinel trap baited with the MB5 blend and CO_2_ outperforms the current golden standard (BG-Sentinel trap with BG-Lure) for monitoring *Ae. aegypti* females and males, in both laboratory and field experiments. The BG-Bowl baited with the MB5 blend is a good candidate for home use. Finally, the results show that CO_2_ is an indispensable component of the attractive blend.
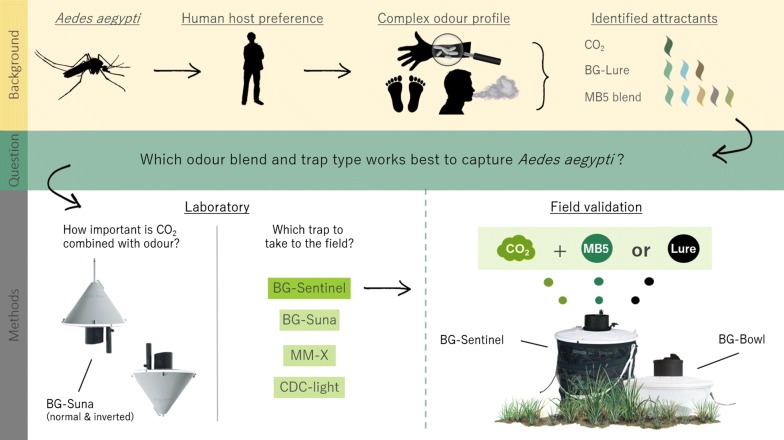

## Background

Arboviral diseases are increasingly threatening human health [1]. Examples of these viruses causing disease are dengue (DENV), chikungunya (CHIKV) and, more recently, Zika virus (ZIKV). These arboviruses have caused considerable numbers of human disease outbreaks in recent years [2]. For example, in Suriname, there were multiple outbreaks caused by DENV between 2008–2016, CHIKV between 2014–2016, and also ZIKV from November 2015 till the end of 2016 [3, 4]. What these arboviruses have in common is that they are predominantly transmitted by the yellow fever mosquito, *Aedes aegypti*. Currently, there are no effective vaccines or medical treatments against infections caused by DENV, CHIKV and ZIKV. Therefore, disease control efforts strongly rely on control of *Ae. aegypti*. Management of (potential) larval habitats, spraying insecticides against adult mosquitoes, using larvicides, and the use of *N,N*-diethyl-*meta*-toluamide (DEET), are common practices to control transmission of *Aedes*-borne diseases. Bednets are less effective since *Ae. aegypti* is a day-biting mosquito. However, control of *Ae. aegypti* is hampered by resistance to multiple classes of insecticides [5–8].

In Suriname, *Ae. aegypti* is mostly found in the northern coastal plain which is the most inhabited area, and which includes the capital city, Paramaribo [9]. A study by Hiwat et al. [9] showed that the Breteau index (the number of *Ae. aegypti* positive water containers per 100 houses) was high in four tested coastal areas, ranging between 105.7–346.6. However, *Ae. aegypti* is increasingly found in inland villages too, probably due to improved infrastructure heading inlands. This is best illustrated by a survey of the Bureau of Public Health (BOG) in response to a female Dutch traveller who probably contracted yellow fever in the inlands in 2017 [10]. During the survey, *Ae. aegypti* was found in the inland village Brownsweg and found at an altitude of 400 metres at the top of the Brownsberg mountain (BOG, unpublished data).

*Aedes aegypti* females play a key role in the transmission of DENV, CHIKV and ZIKV between humans because of their strong preference for human blood as nutritional source (anthropophily) [11]. Olfactory cues are the most important external stimuli that determine this host preference [12]. The olfactory receptors of the mosquito are adapted to respond to specific odours released by the host. Several volatiles that mediate host-seeking behaviour in mosquitoes have already been identified. Carbon dioxide is one of the most important volatiles that signals the presence of a host. It is used as a general cue since it is exhaled by all vertebrates. It causes activation of, and attracts the female mosquito [12–14]. However, carbon dioxide provides little information for anthropophilic mosquitoes that need more specific cues to distinguish between hosts of the same species [12]. The skin or bacteria on the skin excrete these odour cues that guide mosquitoes to their host [15]. Several studies resulted in the production of synthetic blends that attract as many mosquitoes as, and sometimes even more mosquitoes than human odour [16–19]. As a result of these studies, the ‘Mbita blend’ (also known as the MB5 blend) was developed, consisting of five different volatile compounds: lactic acid, ammonia, tetradecanoic acid, 3-methyl-1-butanol and butan-1-amine [19, 20]. The MB5 blend has been developed and optimised for attracting African anthropophilic *Anopheles* mosquitoes towards the BG-Suna trap [16, 19, 21, 22]. Interestingly, the blend, when combined with CO_2_, also effectively caught *Ae. albopictus* during a field study in Italy in the BG-Sentinel trap [17]. An alternative odour blend is the commercially available BG-Lure (Biogents, Regensburg, Germany). This blend contains ammonia, (S)-lactic acid, and hexanoic acid. The effectiveness of BG-Lure has previously been shown when used in the BG-Sentinel trap, for collecting *Ae. aegypti*, *Ae. albopictus* and other species in tropical areas [23, 24], North America [25] and Europe [26, 27], especially in combination with releasing CO_2_. Several studies have shown that adding CO_2_ to traps increases mosquito catches [28, 29].

Based on these findings, new options for *Ae. aegypti* monitoring and surveillance can be explored. Blends of synthetic chemical attractants can be used to attract host-seeking mosquitoes, and with that, mass mosquito trapping systems can possibly be developed. The principle of such a mass-trapping system was evaluated for malaria vectors in a large field study on Rusinga Island, Kenya [30]. On this island, mass-coverage of 4358 households with the BG-Suna trap baited with the MB5 blend and 2-butanone, a CO_2_ replacement [31], led to a reduction in *Anopheles funestus* populations, the main malaria vector on the island. Moreover, it led to an overall reduction of 29.8% malaria prevalence [30]. Although developed to reduce malaria, these odour-baited traps might also offer a solution for *Aedes-*borne diseases, which are prevalent in Suriname and other South American countries. The present study aimed to optimize several odour-baited trap types in the laboratory, followed by testing the best combinations of trap and odour bait in the field with the aim to improve *Ae. aegypti* monitoring and surveillance techniques.

## Methods

First, the BG-Suna trap was optimised in the laboratory by testing different positions that affect air and thus odour flow around the trap. Secondly, the MB5 blend, CO_2_, and a combination of both was tested for their attractiveness to laboratory-reared *Ae. aegypti* in screen cage tests. Thirdly, we tested the attractiveness of different trap types (BG-Suna, BG-Sentinel, MM-X and CDC light trap) in the same screen cage set-up. Finally, we evaluated laboratory results in the field in Paramaribo, Suriname, by using different combinations of the MB5 and BG-Lure odour blends, CO_2_ and two trap types (BG-Sentinel and BG-Bowl) using a Latin Square design.

### Optimisation of traps and odour blends in the laboratory

#### Laboratory-reared mosquitoes

Laboratory-reared *Ae. aegypti* mosquitoes (Rockefeller strain, Bayer, Germany) were used for all experiments performed at the Laboratory of Entomology, Wageningen University and Research, Wageningen, The Netherlands. Adult mosquitoes were maintained in 30 cm cubic rearing cages in a climate-controlled room at a temperature of 27 ± 1 °C, a relative humidity of 75 ± 5%, and a 12:12 h light:dark photoperiod. Adult mosquitoes were fed *ad libitum* on 6% glucose solution. The mosquitoes were blood-fed three times a week on human blood (Sanquin Blood Supply Foundation, Nijmegen, The Netherlands) during the light period. The blood was offered through Parafilm® (Bemis NA, Neenah, USA) using a Hemotek® PS5 membrane feeding system equipped with FU1 feeders (Discovery Workshops, Accrington, UK) at 37 °C. Adult mosquitoes laid their eggs on moist filter paper placed in a cup of tap water. The eggs were dried for 3–4 days and then placed in trays containing tap water with three drops of Liquifry No. 1 (Interpet, Dorking, UK). The larvae were fed twice a week with TetraMin® Baby fish food (Tetra Werke Company, Melle, Germany). The newly emerging adults were placed in rearing cages or cages for experimental use.

#### Odour and CO_2_ production

The MB5 blend, designed to mimic human odour, was tested for its attractiveness for *Ae. aegypti* in the laboratory. The MB5 blend contains ammonia, (S)-lactic acid, tetra decanoic acid, 3-methyl-1-butanol and butan-1-amine in specific concentrations, which are impregnated on nylon strips as previously described by Verhulst et al. [32]. All strips were handled with clean latex gloves and stored in aluminium foil and a zip bag at − 20 °C to prevent contamination with other odours. In the laboratory experiments, 5% CO_2_ was added to the traps from a pressurized gas cylinder (Linde Gas Benelux B.V., Schiedam, The Netherlands) at a flow rate of 250 ml/min. The flow rate was regulated by a flow meter (Sho-Rate model GT1355; Brooks Instruments, Ede, The Netherlands).

#### Experimental design

In order to select the most optimal combination of trap type and odour-bait prior to validation in the field, four different experiments were performed in large, screened cages that allowed free-flying mosquitoes to choose their preferred trap. For this purpose, experiments were performed in a cage of 290 × 250 × 250 cm (Howitech, Bolsward, The Netherlands) inside a climate-controlled room (temperature 27 ± 1.5 °C, relative humidity 70 ± 4 %) under light conditions between 08:00 h and 16:00 h. Before and after each experiment and in between treatments, the traps were cleaned with 70% ethanol. During experiments, surgical gloves were worn to prevent contamination with human odour. Unfed, 4–9 day-old female *Ae. aegypti* mosquitoes were placed in cylindrical release cages (11 cm in diameter, 12.5 cm in height), 17–20 h prior to the experiments. During that time mosquitoes were provided with tap water only from damp cotton wool.

**Fig. 1:**
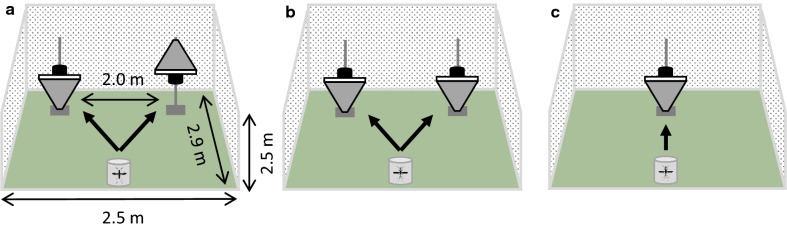
Position of traps in screen cage experiments. **a** Schematic set-up of Cage experiment 1, testing the attractiveness of the BG-Suna trap in the normal (right) *versus* inverted (left) position. Both traps are kept in place using a metal stand. **b** Set-up of both Cage experiment 2, testing the attractiveness of the MB5 blend and CO_2_ compared to an unbaited trap, and 3, testing the attractiveness of the MB5 blend and CO_2_ compared to each other. **c** Set-up of Cage experiment 4, single choice assays to compare four different trap types

*Cage experiment 1: attractiveness of the BG-Suna trap in normal and inverted position.* A previous study performed by Verhulst et al. [32] showed that *Ae. aegypti* can be attracted to a BG-Suna trap when baited with the MB5 blend and CO_2_. The air dynamics surrounding the BG-Suna trap differs when the traps are placed in the normal hanging position compared to the standing inverted position, causing different flight patterns and capture dynamics in *An. coluzzii* mosquitoes [33]. To optimise the trapping efficacy of the BG-Suna trap (Biogents) for *Ae. aegypti*, we tested these normal and inverted positions (Fig. 1a) directly against each other by placing each trap in a corner equidistant and opposite to the side of the release cage where mosquitoes were released. The BG-Suna trap was suspended from a metal stand at 30 cm above ground level measured from the perforated base of the BG-Suna trap [34]. The inverted trap was placed on the ground (Fig. 1a). The traps were both baited with MB5 and CO_2_. Eight replicates of 75 mosquitoes per replicate were performed over two days.

*Cage experiment 2: attractiveness of the MB5 blend and CO*_*2*_*compared to an unbaited trap.* In the previous experiment we determined that the inverted BG-Suna trap outperformed the trap in the normal position. Thus, to determine the attractiveness of the MB5 blend, CO_2_, and the added value of using both cues, unbaited inverted BG-Suna traps were placed against odour-baited inverted BG-Suna traps (Fig. 1b) with the following four treatments: (i) no cues; (ii) CO_2_ alone; (iii) MB5 blend alone; and (iv) CO_2_ + MB5. Per treatment, four replicates of 75 mosquitoes per replicate were performed over two days.

*Cage experiment 3: attractiveness of the MB5 blend and CO*_*2*_*compared to each other.* Inverted odour-baited BG-Suna traps were directly tested against each other (Fig. 1b) with the following three treatments: (i) CO_2_; (ii) MB5 blend; and (iii) CO_2_ + MB5 blend. Per treatment, four replicates of 75 mosquitoes per replicate were performed over two days.

*Cage experiment 4: trap type comparison.* To assess the best trap type for catching *Ae. aegypti*, the trapping efficacy of the inverted BG-Suna trap, the BG-Sentinel 2 trap (Biogents), the MM-X trap (American Biophysics Cooperation, North Kingstown, RI, USA), and the CDC-light trap (John W. Hock Company, Gainesville, FL, USA) was compared. The BG-Sentinel trap is described as the gold standard for *Aedes* surveillance, using visual and olfactory cues and outperforming many other traps [24, 35]. The MM-X trap proved to be very effective for trapping *An. gambiae* [36], but also for *Ae. aegypti* [37]. The fourth trap type included in the experiment was the CDC-light trap. This trap is actually not an odour-baited trap, but it is based on the use of light to attract mosquitoes. It includes a fan to draw mosquitoes into the trap. This trap can also be used without light, depending on the mosquito species [38]. For diurnal mosquitoes such as *Ae. aegypti* the light is not necessary, and therefore not used in this set-up. When using mosquito traps, the height at which the traps are placed should be considered [39]. The BG-Suna trap was suspended from a metal stand at 30 cm above ground level measured from the perforated base of the BG-Suna trap [34]. The BG-Sentinel trap was placed on the ground, with the capture opening at 40 cm height. The MM-X trap was suspended from a metal stand at 15 cm above ground [40], and the CDC-light trap was also suspended from a metal stand, at 50 cm above ground. Traps were individually tested in single-choice experiments (Fig. 1c). Each trap was tested eight times over four different days with 75 mosquitoes released per test. All traps were baited with the MB5 blend and CO_2_.

All four cage experiments lasted 15 min, thereafter the release cage and traps were closed, and the mosquitoes that were not caught in a trap were removed using a vacuum cleaner. Trapped mosquitoes were counted after they were knocked down by placing the trap in a large freezer for 5–10 min. At the beginning of each experiment, temperature and relative humidity were measured using a Tinytag Ultra data logger (model TGU-1500; INTAB Benelux, Cuijk, The Netherlands). The sequence of tested traps was randomized per day to avoid potential bias as a result of the time of testing.

### Validation of traps and odour combinations in the field

#### Study site in Paramaribo

**Fig. 2:**
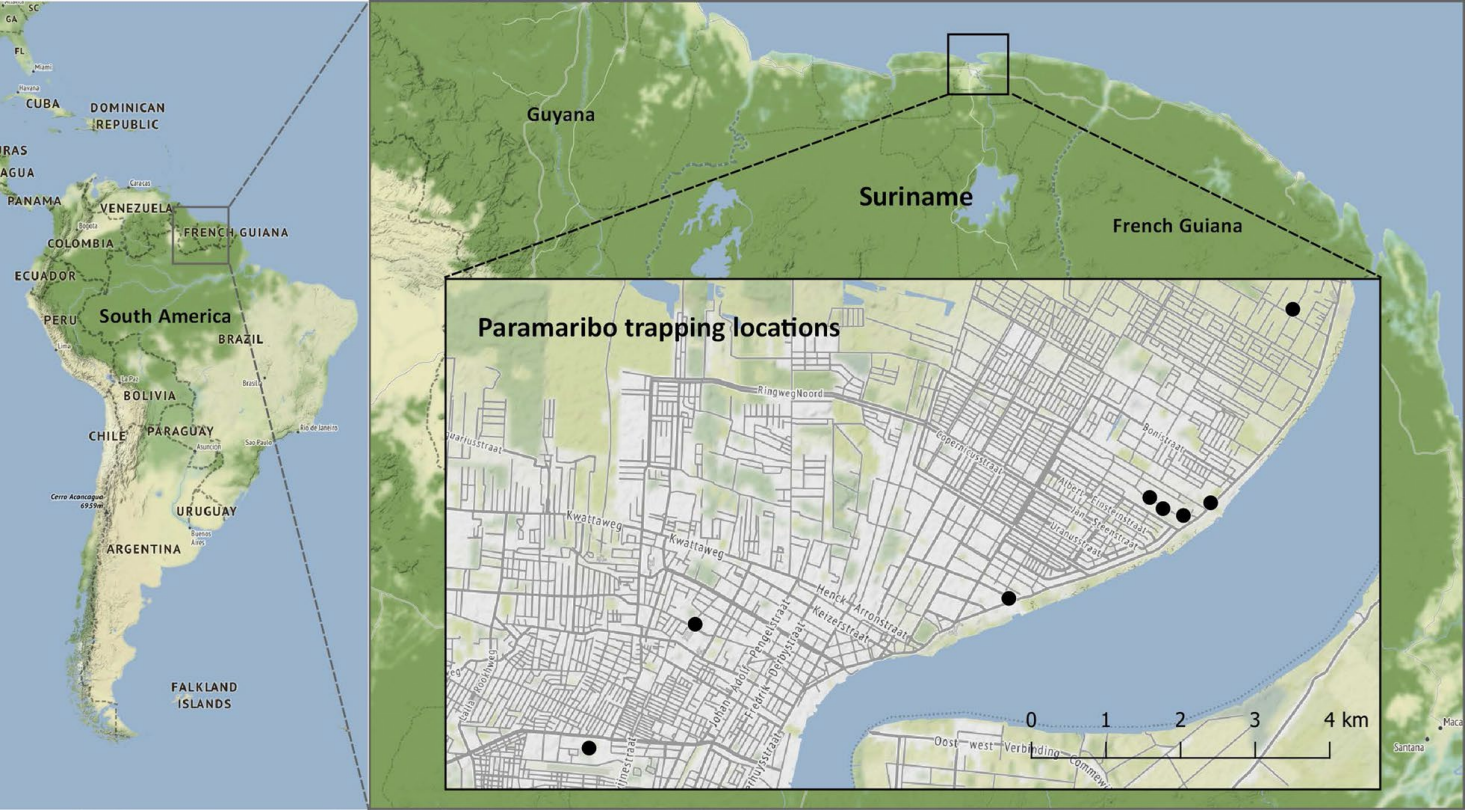
Maps displaying the eight trapping locations in Paramaribo, Suriname. Each study location is shown with a black dot. The trap study focussed on the Paramaribo district. The map was made using QGIS (version 3.4.3 ‘Madeira’) with map data from OpenStreetMap contributors (www.qgis.org and www.openstreetmap.org)

The field study was conducted in the district of Paramaribo, the most populated district of Suriname. In 2012, the Paramaribo District had an estimated population of 240,924 inhabitants and an estimated 182 inhabitants per square kilometre [41]. Paramaribo is the capital city of the Paramaribo District and of Suriname (Fig. 2). The climate of Suriname can be described as a tropical rainforest climate, type Af of the Köppen–Geiger climate classification system [42, 43]. Field experiments with the odour-baited traps were performed from March till June 2017. This was during the short dry season which ranges from the end of January to late April, and the long rainy season which ranges from the end of April to mid-August. Average annual rainfall in Paramaribo is 2200 mm, and the mean temperature is 27.1 °C [43]. Prior to the actual trap studies, we searched for suitable locations where *Ae. aegypti* was present by setting up oviposition and BG-Sentinel traps in the areas of interest to monitor mosquito activity. Finally, we selected eight locations with a distance of at least 200 m distance between them (Fig. 2). Traps were placed inside a home or building with a connection to the outside, *via* tropical windows or *via* open doorways, an open garage or roofed patio.

#### Odour and CO_2_ production

For the field experiments the MB5 blend and the BG-Lure (Biogents) were used. The MB5 blend was produced and stored as mentioned earlier in the laboratory method section of this paper. The BG-Lure consists of ammonia, (S)-lactic acid, and hexanoic acid and is applied on granules in a plastic cartridge. To make the experiment feasible in Suriname we had to switch to a different CO_2_ source, and thus made the practical decision to use CO_2_ made by fermentation. Carbon dioxide was produced and added to the traps by mixing 250 g sugar (Demerara Cane Sugar, The Guiana Sugar Corporation Inc., Ogle estate, Guyana), 17.5 g yeast and 2 l water in a 4-litre jerry can [29]. A silicone tube (⌀7 mm; Rubberbv, Hilversum, The Netherlands) fixed in an opening of the lid of the can was connected with the CO_2_ release opening of the trap. The mixture was replaced every trapping day. During the first field experiment ‘Bruggeman instant yeast blue’ (Algist Bruggeman, Gent, Belgium), was used. For the second field experiment the same yeast was not available, therefore we used ‛Fleischmann’s Instant Yeastʼ (ACH Food Companies Inc., Memphis, Canada).

#### Experimental design

Based on our laboratory results, we set out to determine the attractiveness of the MB5 blend and CO_2_ to mosquitoes, and to evaluate the added value of using both cues in the field. Moreover, we aimed to compare the MB5 blend to the commercially available BG-Lure which previously showed to be effective for capturing *Ae. aegypti* and *Ae. albopictus* in tropical areas [23, 24]. In addition, we aimed to test a new prototype mosquito trap called the BG-Bowl (Biogents; for trap image see Additional file 1: Figure S1). The major limiting factor in deploying the BG-Sentinel trap in area-wide control programs are the costs per trap. For this purpose, Biogents designed a new and cheaper trap, the BG-Bowl. The BG-Bowl uses the same counter-flow system to suck in mosquitoes and expel odour but is smaller than the BG-Sentinel trap. The traps were placed in the morning between 9:00–10:30 h and emptied after 24 h. The experiments were designed as 4 × 4 Latin squares which allowed for blocking into two directions, in this case by location and day. A data logger (Tinytag Plus 2, model TGP-4500; Gemini Data Loggers Ltd, Chichester, UK) was placed in the city to record temperature and humidity every 10 min for the duration of each trapping day. When handling the traps, latex gloves were used to avoid odour contamination. The odour baits remained in the assigned traps, and traps were rotated among the different sampling locations.

*Field experiment 1: evaluation of the attractiveness of the MB5 blend and CO*_*2*_. To evaluate the attractiveness of the MB5 blend, CO_2_, and their combined effect, BG-Sentinel traps were used with the following four treatments: (i) no cues; (ii) CO_2_; (iii) MB5 blend; and (iv) CO_2_ + MB5 blend. The experiment was replicated twice in space and three times in time, resulting in eight trapping locations (Fig. 2) and 12 trapping days (see Latin square design in Additional file 2: Table S1).

*Field experiment 2: evaluation of two mosquito traps and two different odour blends.* The BG-Sentinel trap was compared with the BG-Bowl and both traps were baited with CO_2_ and either the MB5 blend or the BG-Lure to attract mosquitoes. The four tested treatments were: (i) BG-Sentinel + MB5; (ii) BG-Sentinel + BG-Lure; (iii) BG-Bowl + MB5; and (iv) BG-Bowl + BG-Lure. The BG-Lure cartridge was added to the trap *via* the opening in the lid according to the manufacturer’s instruction manual. The MB5 blend was added by placing a paperclip inside the trap at the same place of the BG-Lure, on which the odour-baited nylon sock could be tied. Since the previous experiment showed the importance of CO_2_, it was added to all treatments. Carbon dioxide was added by inserting the CO_2_ source *via* a silicon tube (⌀7 mm; Rubberbv) in the trap. The BG-Bowl had to be adjusted slightly for this study, since a pilot test showed that collected mosquitoes could escape the trap *via* the drainage openings in the bottom. Therefore, the gaps were covered with grey duct-tape. There was no input place for CO_2_ in the BG-Bowl so a small hole was drilled on the side in which the tube fitted tightly (Additional file 1: Figure S1). The experiment was based on a 4 × 4 Latin square design and was replicated twice in space and twice in time (see Latin square design in Additional file 2: Table S2), resulting in eight trapping locations (Fig. 2) and eight trapping days.

The trapped mosquitoes were collected and placed in a − 20 °C freezer for 10 min. In case of the BG-Bowl, the whole trap was placed at − 20 °C since it was not possible to get the mosquitoes out otherwise. Female and male mosquitoes were counted and identified to the genus and species level if possible, using the South and Central America identification key by Becker et al. [44]. Thereafter, the *Ae. aegypti* females were stored at − 80 °C in Eppendorf tubes that contained silica gel grains and a small tray of Whatman filter paper to prevent water damage and degradation of the mosquitoes. They were sent to the Animal Health Laboratory, ANSES (Maisons-Alfort, France) for virus detection, see reference [45] for more details on the findings.

#### Statistical analysis

The laboratory data were analysed by using a generalized linear model (GLM, binomial, logit link function and dispersion estimated) to test for differences in ratio of mosquitoes caught and trap entry response rates of the mosquitoes towards CO_2_ and odour blends, and different trap types [32]. The ratio was calculated as the number of mosquitoes caught in the treatment trap divided by the total number of mosquitoes caught in both traps. The trap entry response ‘R’ is expressed as the total number of mosquitoes caught in both treatments divided by the total number of released mosquitoes. Covariates associated with the experimental design (mosquito age, day and time of mosquito release, temperature, relative humidity, airflow and trap position) were tested but removed from the model when not significant (*P* > 0.05). In laboratory experiments 2 and 3 we used the 95% CI of the predicted proportion of mosquitoes choosing a specific treatment, derived from the GLM, to assess if mosquito choice differed significantly from a 50:50 distribution [46].

The field data were analysed using a different GLM (negative binomial, log function and dispersion estimated) to test the difference in trapping effectiveness of the different odour baits and traps (BG-Sentinel and BG-Bowl). *Aedes aegypti* and *Culex* spp. mosquitoes, and captured females and males were analysed separately. Main effects tested were the odour treatments and trap types. Covariates associated with the experimental design (location, day, week blocks, temperature and humidity) were tested but removed from the model when not significant (*P* > 0.05). All possible two-way interactions on the number of mosquitoes captured were also added to the GLM and non-significant factors were removed from the model.

Given a set of candidate models for the laboratory and field data, the preferred model was the one with the lowest AIC value. The final model, including significant covariates, was used to calculate the estimated mean trap catches and standard errors [17]. When differences were found, we performed pair-wise comparisons to indicate differences between the means (LSD correction). All analyses were performed using SPSS statistical software (version 25, IBM Corporation, New York, USA). Before performing the analyses, outliers were identified using boxplots and their nature was investigated, but they were not removed. Moreover, data were checked for normality and effects for all tests were considered significant when *P* < 0.05.

## Results

### Optimisation of traps and odours in the laboratory

#### Cage experiment 1: attractiveness of the BG-Suna trap in normal and inverted position

**Fig. 3:**
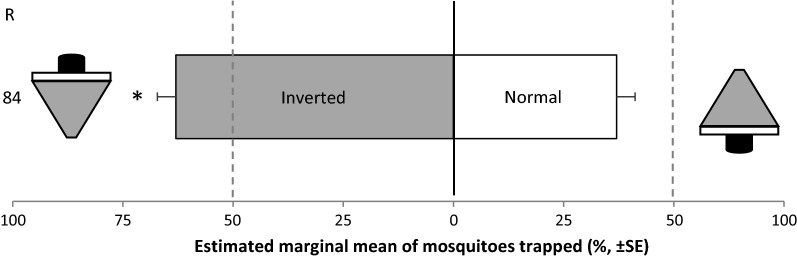
*Aedes aegypti* caught in the inverted BG-Suna trap compared to the BG-Suna trap in normal position. Eight replicates of 75 mosquitoes each were performed. Estimated marginal mean percentages are presented, assuming a binomial distribution and logit link function. Error bars represent standard errors of the mean. The asterisk (*) indicates a significant difference, *P* < 0.05 (GLM). There were no covariates included in the final GLM model. The trap entry response ‘R’ is expressed as the number of mosquitoes caught in both treatments divided by the number of released mosquitoes

The attractiveness of the BG-Suna trap’s position was investigated by comparing the inverted with the normal position (Fig. 3). Of the 600 *Ae. aegypti* released the mean trap entry response ‘R’ was 84 ± 2.3% (standard error, SE). The inverted BG-Suna trap caught 63 ± 4.2% of all mosquitoes trapped, which was significantly more than the 37 ± 4.2% caught in the BG-Suna trap in normal position (GLM: *df* = 7, *P* = 0.003, see Additional file 3: Table S3, for uncorrected means).

#### Cage experiment 2: attractiveness of the MB5 blend and CO_2_ compared to an unbaited trap

**Fig. 4:**
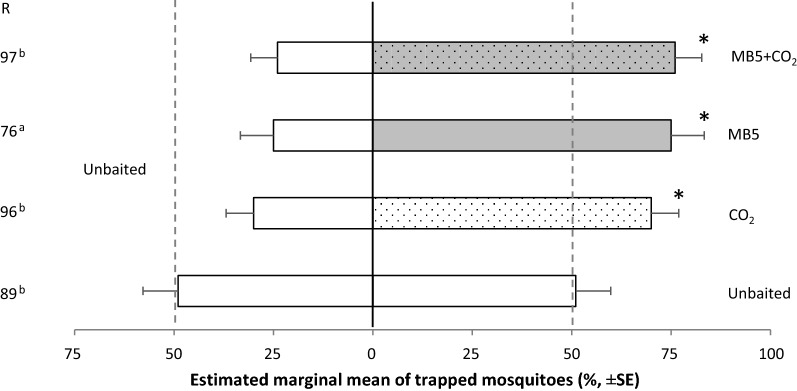
*Aedes aegypti* caught in an unbaited inverted BG-Suna trap compared to a BG-Suna trap baited with CO_2_, MB5 or MB5 + CO_2_. Per treatment, four times 75 mosquitoes were released. Estimated marginal mean percentages are presented, assuming a binomial distribution and logit link function. Error bars represent standard errors of the mean. The asterisk (*) indicates a significant difference from a 50:50 ratio *P* < 0.05 (GLM: 95% CI). There were no significant covariates included in the final GLM model. The trap entry response ‘R’ is expressed as the number of mosquitoes caught in both treatments divided by the number of released mosquitoes. Significant differences in ‘R’ are indicated with letters a and b (GLM, LSD: *P* < 0.05)

The effect of the MB5 blend and CO_2_ on capture rates of *Ae. aegypti* were tested using inverted BG-Suna traps in the screen cage assay (Fig. 4). As a control, two unbaited BG-Suna traps were tested against each other. As expected, the MB5 + CO_2_, MB5 and CO_2_ treatments significantly increased trap captures in comparison to a trap without any attractant (GLM: *df* = 12, 95% CI, respectively: 0.61–0.87, 0.56–0.88 and 0.55–0.81, *P* < 0.05), while equal numbers were caught on both sides when using two unbaited control traps (GLM: *df* = 12, 95% CI: 0.34–0.67, *P* > 0.05). The trap entry response ‘R’ did only differ for the MB5 treatment, showing a significantly lower response rate than all other treatments (GLM: *df* = 12, *P* < 0.03). See Additional file 3: Table S4, for uncorrected means.

#### Cage experiment 3: attractiveness of the MB5 blend and CO_2_ compared to each other

**Fig. 5:**
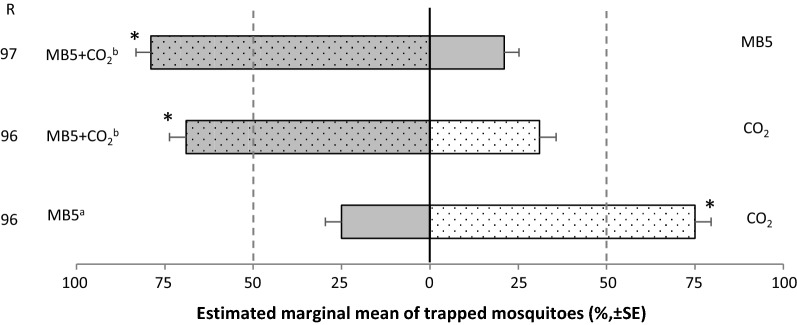
*Aedes aegypti* caught in inverted BG-Suna traps baited with CO_2_, MB5 or MB5 + CO_2_ in dual-choice experiments. Per treatment, four times 75 mosquitoes were released. Estimated marginal mean percentages are presented, assuming a binomial distribution and logit link function. Error bars represent standard errors of the mean. The asterisk (*) indicates a significant difference from a 50:50 ratio (GLM: 95% CI, *P* < 0.05). The letters a and b indicate differences between means of the treatments (GLM, LSD: *P* < 0.05). Relative humidity was included in the GLM model as covariate (*P* < 0.001). The trap entry response ‘R’ is expressed as the number of mosquitoes caught in both treatments divided by the number of released mosquitoes. There were no significant differences in ‘R’ (GLM, LSD: *P* > 0.05)

Next, the MB5 blend, CO_2_, and MB5 + CO_2_ were tested directly against each other to determine their relative effect on *Ae. aegypti* capture rates (Fig. 5). The traps baited with MB5 + CO_2_ caught significantly more mosquitoes compared to the traps baited only with MB5 or CO_2_ (GLM: *df* = 8, 95% CI, respectively 0.69–0.86 and 0.59–0.77, *P* < 0.05). The MB5 + CO_2_ treatment caught 79 ± 4.2% (SE) of all mosquitoes trapped when compared to the MB5 treatment, and 69 ± 4.7% of all mosquitoes trapped when compared to the CO_2_ treatment. When MB5 was tested against CO_2_, the MB5 treatment only caught 25 ± 4.6% of all mosquitoes trapped, which was significantly lower than the 75 ± 4.6% caught with the CO_2_ treatment (GLM: *df* = 8, 95% CI: 0.17–0.35, *P* < 0.05). Moreover, pairwise comparisons showed that the trapping efficacy of the MB5 +  CO_2_ treatment against either CO_2_ or MB5 were not different from each other (GLM, LSD: *df* = 8, *P* > 0.05). However, both did differ from the MB5 treatment against CO_2_ (GLM, LSD: *df* = 8, *P* < 0.001). Relative humidity significantly affected trap captures and was therefore included in the final model (GLM: *df* = 1, *P* < 0.001). The trap entry response ‘R’ of the different treatments did not differ from each other (GLM: *df* = 9, *P* > 0.05). See Additional file 3: Table S5, for uncorrected means.

#### Cage experiment 4: trap type comparison

**Fig. 6:**
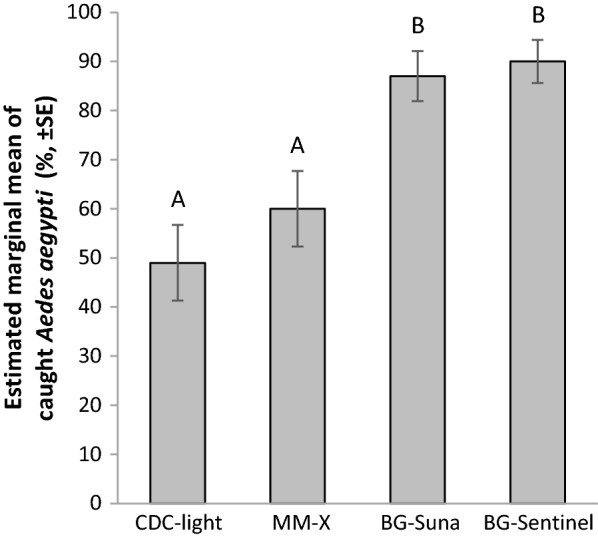
Percentage of *Aedes aegypti* caught in the CDC-light trap, the MM-X trap, the BG-Sentinel trap and the inverted BG-Suna trap, all baited with MB5 and CO_2_. Per treatment, eight times 75 mosquitoes were released. Estimated marginal mean percentages are presented. Error bars represent the standard errors of the mean. Significant differences in trapping efficacy between treatments are indicated with different letters (A and B; GLM, LSD: *P* < 0.001). Relative humidity was included as covariate in the final GLM model (*P* < 0.05)

The next step was to evaluate how trap type affects *Ae. aegypti* capture rates if these traps are baited with the most effective attractants, i.e. MB5 and CO_2_ (Fig. 5). For this purpose, the efficacy of the BG-Sentinel trap, the MM-X trap, CDC-light trap (without light), and the inverted BG-Suna was assessed in single choice assays in the screen cage set-up. The BG-Sentinel trap and the BG-Suna trap performed equally, capturing significantly more *Ae. aegypti* females than the CDC-light trap (GLM: *df* = 27; LSD, for both traps *P* < 0.001, Fig. 3) and MM-X trap (respectively *P* < 0.001 and *P* < 0.003, Fig. 6). Relative humidity significantly affected trap captures and was included in the final model (GLM: *df* = 1, *P* < 0.05). See Additional file 3: Table S6, for uncorrected means. From a practical point of view, the BG-Sentinel was taken to the field for further validation of its trapping efficiency in combination with selected odour baits.

### Field experiments

Overall, a total of 10,425 mosquitoes were collected during the 23 trapping days of this study: 50.4% of these mosquitoes were *Ae. aegypti* and 49.6% were *Culex* spp. A check showed that these culicines were mostly *Culex quinquefasciatus* and *Cx. nigripalpus*. Very rare were specimens of *Mansonia dyari*, *Haemagogus janthinomys* and *Ochlerotatus scapularis*: we collected only one female of each of these species. Noteworthy is the difference in male/female ratio found for *Ae. aegypti* and the culicine species. We caught slightly more *Ae. aegypti* males (55.6%) than females (44.4%) and caught more females of *Culex* spp. (78%) than males (22%).

#### Field experiment 1: evaluation of attractiveness of a synthetic host odour blend and CO_2_

**Fig. 7:**
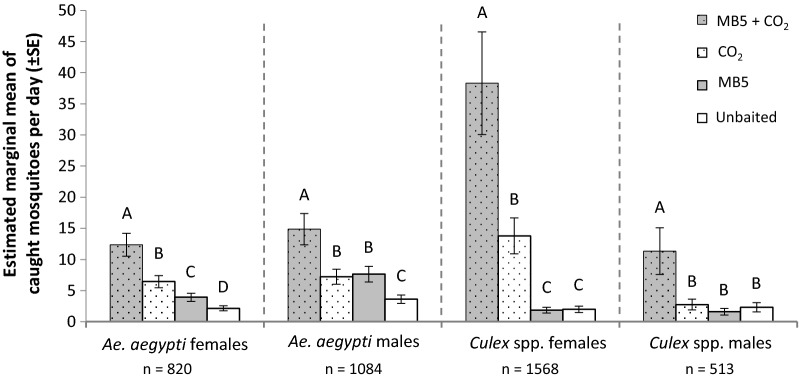
Attraction of *Aedes aegypti* and *Culex* spp. mosquitoes to traps baited with MB5 and/or CO_2_. Estimated marginal mean (GLM) of mosquitoes caught using the BG-Sentinel traps with no blend (unbaited), MB5 blend, CO_2_, and CO_2_ + MB5 blend combined. Error bars represent the standard error of the mean. For each mosquito species and sex: different letters above bars indicate significant differences among treatments (GLM, LSD and *P* < 0.05). See Additional file 4 for additional statistical information

Addition of CO_2_, MB5 or its combination to the BG-Sentinel trap significantly increased trap capture rates of *Ae. aegypti* females (GLM: *df* = 3, *P* < 0.001; Additional file 4: Table S7) in comparison to an unbaited trap. The *post-hoc* test revealed that the most attractive trap was the trap baited with the combination of CO_2_ and MB5, followed by CO_2_ alone and MB5 alone (Fig. 7; Additional file 4: Table S8). Location and week had a significant effect and were included in the final model (GLM: *df* = 7, *P* < 0.001 and *df* = 2, *P* < 0.002, respectively). Captures of *Ae. aegypti* males showed a similar pattern as females, i.e. the trap with CO_2_ and MB5 was most attractive, and the unbaited trap least attractive (GLM: *df* = 3, *P* < 0.001; Additional file 4: Tables S7, S8). Traps baited with only CO_2_ or MB5 did not differ from each other in terms of attractiveness (Fig. 7). Similar to the model for *Ae. aegypti* females, location and week of collection had a significant effect and were included in the final model (GLM: *df* = 7, *P* < 0.001 and *df* = 2, *P* < 0.05, respectively).

For *Culex* spp. females, highest captures were also obtained by using MB5 + CO_2_ (GLM: *df* = 3, *P* < 0.001; Additional file 4: Tables S8, S9). The *post-hoc* LSD test revealed that females were less attracted by CO_2_ alone and least attracted by MB5 alone and unbaited traps (Fig. 7, Additional file 4: Table S7). Trap location was included as a covariate in the final model (GLM: *df* = 7, *P* < 0.001). *Culex* spp. males were also most attracted by MB5 + CO_2_ (GLM: *df* = 3, *P* < 0.001; Additional file 4: Tables S7, S8). The other three treatments did not differ in attractiveness (Fig. 7, Additional file 4: Table S8). Similar to the model for *Culex* females, trap location was included in the final model (GLM: *df* = 7, *P* < 0.001).

It should be noted that four MB5 + CO_2_ data points were excluded from the final analyses because of accidental absence of the odour blend (*n* = 20, Additional file 3: Table S3). One CO_2_ data point was excluded from analysis because of spoilt mosquitoes due to an infestation with ants (*n* = 23, Additional file 3: Table S3). Combining CO_2_ and MB5 had an added effect on all mosquito catches (Fig. 7). Therefore, it was decided to add CO_2_ to the follow-up experiment with different lures and trap types.

#### Field experiment 2: evaluation of two mosquito trap types with two different odour blends

**Fig. 8:**
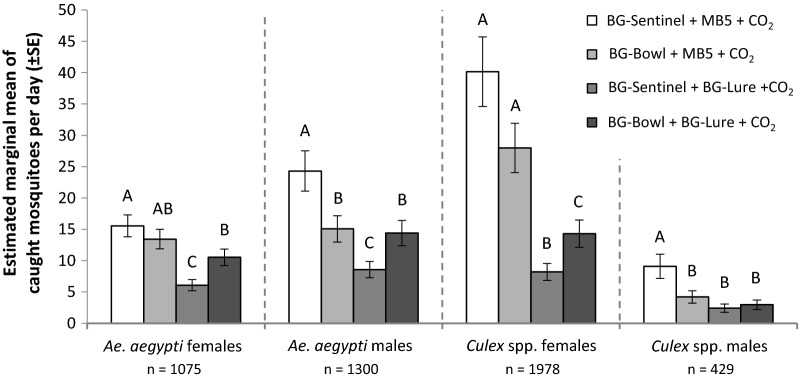
Attraction of *Aedes aegypti* and *Culex* spp. mosquitoes to two different trap types and two different synthetic blends, all with added CO_2_. Estimated marginal means (GLM) of mosquitoes caught using the BG-Sentinel or BG-Bowl trap baited with either the MB5 blend or the BG-Lure. Error bars represent the standard error of the mean (*n* = 16 per treatment). For each mosquito species and sex: different letters above bars indicate significant differences between treatments (GLM, LSD and *P* < 0.05). See Additional file 4 for additional statistical information

For all four groups of mosquitoes analysed (i.e. *Ae. aegypti* males, *Ae. aegypti* females, *Culex* spp. males and *Culex* spp. females) we found an interaction effect between odour blend and trap type (GLM: *df* = 2; *P* = 0.012, *P* = 0.002, *P* = 0.008 and *P* = 0.028, respectively). This interaction suggests that the attractiveness of a specific odour blend depends on the trap type used. More specifically, the *post-hoc* test showed that most *Ae. aegypti* females were caught in the BG-Sentinel and BG-Bowl baited with MB5. This was followed by the BG-Bowl baited with the BG-Lure. Least attractive was the BG-Sentinel baited with the BG-Lure (Fig. 8, Additional file 4: Tables S9, S10). A factor included in the final model was the single effect of the odour blend (GLM: *df* = 1, *P* < 0.001). Location and day were included as covariates in the final model (GLM: *df* = 7, *P* < 0.001; and *df* = 7, *P* < 0.002). The *post-hoc* test for *Ae. aegypti* males shows a similar pattern as for *Ae. aegypti* females. However, most individuals were captured in the BG-Sentinel baited with MB5 (Fig. 8, Additional file 4: Table S10). A factor included in the final model was the single effect of the odour blend (GLM: *df* = 1, *P* < 0.001). Covariates included in the final model were location and relative humidity (GLM: *df* = 7, *P* < 0.001; and *df* = 1, *P* < 0.001).

*Culex* spp. females were most attracted by the BG-Sentinel and BG-Bowl baited with MB5, similar to *Ae. aegypti* females. This was followed by the BG-Bowl baited with the BG-Lure. Least attractive was the BG-Sentinel baited with the BG-Lure (Fig. 8, Additional file 4: Table S10). A factor included in the final model was the single effect of the odour blend (GLM: *df* = 1, *P* < 0.001). Covariates that were included in the final model were location and relative humidity (GLM: *df* = 7, *P* < 0.001; and *df* = 1, *P* < 0.04). Besides the odour treatment and trap type interaction, there was also an interaction between trap type and location for *Culex* spp. males (GLM: *df* = 7, *P* < 0.03). This indicates that the trap performs different not only when baited with a different odour blend but also at different locations. *Post-hoc* test revealed that most males were caught in the BG-Sentinel baited with MB5 and no differences in attraction towards the other three treatments were found (Fig. 8, Additional file 4: Table S10). Covariates that were included in the final *Culex* spp. male model were the single effect of odour blend and location (GLM: *df* = 1, *P* < 0.001; and *df* = 7, *P* < 0.001).

## Discussion

This study shows that the MB5 blend, originally developed for anopheline mosquito species, can be successfully used for capturing *Ae. aegypti*, *Cx. quinquefasciatus* and *Cx. nigripalpus*. Our laboratory study clearly demonstrated that this blend, when used in combination with CO_2_, outperforms the single attractants, i.e. MB5 blend or CO_2_ alone. After observing that the BG-Sentinel trap functioned equally well compared to the BG-Suna trap in our laboratory setting, we investigated the performance of this trap and odour blend in the field in Suriname. In the field study, we confirmed that the combination of the MB5 blend and CO_2_ performs best, as it almost doubles *Ae. aegypti* female captures and more than doubles *Culex* spp. female captures compared to using only CO_2_. In our study the MB5 blend outperformed the commercially available BG-Lure, which was specifically developed for *Ae. aegypti* for use in the BG-Sentinel. Similarly, studies from Owino et al. [47, 48] showed that the BG-Lure is not the most effective bait for *Aedes aegypti* compared to natural human odours and hexanoic acid. Our results and those from Owino et al. [47, 48] challenge the current view on capturing *Aedes* mosquitoes in which the BG-Sentinel trap in combination with the BG-Lure as attractant is considered the gold standard [23–25].

A possible reason for the reduced captures using the BG-Lure is that the airflow of the BG-Sentinel is less optimal for the cartridge design of the BG-Lure and odours are thus expelled more efficiently by the BG-Bowl. Both traps differ substantially in height and material but do have the same type of ventilator. From a practical point of view, the BG-Sentinel remains the most useful trap type for monitoring and surveillance purposes because of the catch-bag that is placed before the fan which does not damage collected mosquitoes as opposed to the BG-Bowl [49]. Yet, the BG-Bowl can be a cheaper alternative that can be used by homeowners for prevention and control purposes. Nevertheless, there are a few minor alterations that probably should be made to the BG-Bowl. The used version of the BG-Bowl in this study does not allow for the insertion of an external CO_2_ source, and the rainwater drainage openings are too large, allowing *Ae. aegypti* mosquitoes to escape the trap *via* these openings.

The study showed that CO_2_ is an indispensable part of the odour blend mixture. During the field study CO_2_ was made using yeast fermented sugar. The use of this method in the field is advantageous because of its straightforward protocol and readily available ingredients [50]. However, the yeast, sugar and water mixtures need to be replaced every day to produce sufficient CO_2_ to capture mosquitoes. Alternative ways of making CO_2_ are using heavy and expensive gas cylinders, or dry ice which is hard to obtain in the tropics [51, 52]. For mass-trapping of *Ae. aegypti*, a cheaper and long-lasting method is desired. A candidate compound to replace CO_2_ is 2-butanone which, tested in combination with the MB5 blend, was equally attractive as CO_2_ (produced with yeast fermented molasses) with the MB5 blend for *An. funestus* and *An. gambiae* (*s.l.*) mosquitoes [31]. However, for other mosquito species including *Culex* spp. it was less attractive.

Besides catching female mosquitoes, we also caught a considerable number of *Ae. aegypti* males. Other studies also report capturing *Ae. aegypti* and *Ae. albopictus* males using BG-Sentinel traps baited with CO_2_, MB5 blend or BG-Lure [17, 23, 47, 48, 53]. These findings are not surprising. It was previously shown that *Aedes* males respond to host-odours [54] in order to intercept host-seeking females near a potential host [55]*. Culex* spp. do not show this type of mating behaviour, which may explain the lower trap catches of males of this genus in comparison with females. Even though it was not the original goal of the study, data on the population dynamics of male *Ae. aegypti* are very useful for optimising sterile insect techniques [56]. Knowledge on survival, dispersal and longevity of these males is important for the success of this control strategy [57]. In addition, *Ae. aegypti* males display protandry [58], and thus emerge before females do. Therefore, male catches may also provide information on female emergence [53].

The *Culex* spp. caught in our traps in Suriname consisted of *Cx. quinquefasciatus* and *Cx. nigripalpus*. In Suriname, both are considered nuisance species and not medically important. Besides catching medically relevant species, the odour-baited traps may potentially also be used to reduce mosquito nuisance. Particularly *Cx. quinquefasciatus* can be highly abundant. Yet, it remains important to keep track of nuisance species. *Culex quinquefasciatus* can in fact transmit lymphatic filariasis [59], which has been eliminated from Suriname since 2011. However, there is still a risk of importing lymphatic filariasis from neighbouring Guyana, where it is still reported as being endemic by the WHO [60]. Next to that, the hypothesis was put forward that *Culex* species can contribute to ZIKV transmission, because of the relatively low vector competence of *Aedes* mosquitoes to ZIKV and, therefore explain the unexpected rapid spread of ZIKV in the Americas [61]. However, the study of Fernandes et al. [61] showed that experimentally infected *Cx. quinquefasciatus* mosquitoes from Rio de Janeiro, Brazil, were not able to transmit ZIKV. Moreover, besides being incompetent to transmit ZIKV in the laboratory, no naturally ZIKV infected *Cx. quinquefasciatus* mosquitoes have been found in the Americas to date [61].

Our study shows that the BG-Sentinel baited with the MB5 blend and CO_2_ is highly suitable for monitoring *Ae. aegypti* mosquitoes. One of the questions that remains, however, is to which extent odour-baited traps can also be used in vector control and hence in reducing disease transmission. The study of Homan et al. [30] showed that mass trapping of anopheline mosquitoes with an MB5-baited BG-Suna trap reduced malaria prevalence with 30% in Kenya. Nonetheless, several questions still remain for *Ae. aegypti* such as to which extent a trap works efficiently at a household level or what coverage is needed to reduce *Ae. aegypti* populations? In case of the day biting *Ae. aegypti* mosquito that is active when most people are at home, it might be necessary to focus vector control with traps on, for example, workplace areas. Upon recommendation from the BOG staff, the traps during this study were placed indoors with a connection (for example an open window) to the outdoors or in a shaded spot outside. Factors that can influence capture rates are, amongst others, placement inside or outside houses, temperature, relative humidity, shadow or sun, and rainfall. A study of Crepeau et al. [62] showed that the BG-Sentinel trap caught over three times more *Ae. albopictus* mosquitoes when placed in a shady area compared to a sunny area. This could strongly impact estimates on mosquito population size and hence have an effect on vector control decisions. Such future studies should go hand in hand with social sciences, i.e. measuring customer experiences and rolling out community engagement programs, to safeguard effective implementation of interventions and create public support.

## Conclusions

Our study demonstrated the effectiveness of traps baited with the MB5 blend and CO_2_ for monitoring *Ae. aegypti* females and males, through both laboratory and confirmative field experiments. Interestingly, the MB5 blend outperformed the current golden standard, the BG-Lure applied in the BG-Sentinel trap. The BG-Bowl trap also attracted *Ae. aegypti* when baited with either the MB5 blend or the BG-Lure and is a good candidate for home use. Furthermore, the results show that CO_2_ is an indispensable component of the attractive blend. To implement odour-baited traps for control purposes, more research on alternatives for CO_2_ production is needed, and other factors, such as the effect of trap placement on mosquito catches, should be further explored. Outbreaks of emerging arboviruses like Zika, dengue and chikungunya emphasize that it is of paramount importance to keep investing in novel technologies for vector-borne disease control.

## Supplementary information

**Additional file 1: Figure S1.** The BG-Bowl used in the study.

**Additional file 2: Table S1.** The 4 × 4 Latin Square design used in Field experiment 1. **Table S2.** The 4 × 4 Latin Square design used in Field experiment 2.

**Additional file 3: Table S3.** Uncorrected means (%, ± SE) of cage experiment 1. **Table S4.** Uncorrected means (%, ± SE) of Cage experiment 2. **Table S5.** Uncorrected means (%, ± SE) of Cage experiment 3. **Table S6.** Uncorrected means (%, ± SE) of Cage experiment 4.

**Additional file 4: Table S7.** Field experiment 1, mean (± SE) of mosquitoes caught per treatment per day. **Table S8.** Field experiment 1, *P*-values of pairwise comparisons (GLM) after LSD correction. **Table S9.** Field experiment 2, mean (± SE) of mosquitoes caught per treatment per day. **Table S10.** Field experiment 2, *P*-values of pairwise comparisons (GLM) after LSD correction.

## Data Availability

Data supporting the conclusions of this article are included within the article and its additional files. The datasets used and/or analysed during the present study are available from the corresponding author upon reasonable request.

## Abbreviations

AIC: Akaikeʼs information criterion
BOG: Bureau of Public Health, Suriname
CDC light trap: Centre for Disease Control light trap
CHIKV: chikungunya virus
CI: confidence interval
DEET: diethyltoluamide
DENV: dengue virus
GLM: generalized linear model
LSD: least significant difference
MB5 blend: Mbita blend consisting out of five components
MM-X trap: mosquito magnet-X trap
R: trap entry response, defined in methods
SE: standard error of the mean
ZIKV: Zika virus
