# Supplementary material for: Optimisation and field validation of odour-baited traps for surveillance of Aedes aegypti adults in Paramaribo, Suriname
Source: Parasit Vectors. 2020 Mar 6;13:121. doi: 10.1186/s13071-020-4001-y (PMC7059684; doi:10.1186/s13071-020-4001-y)
Supplement: Supplementary file 1 — Additional file 1: Figure S1. The BG-Bowl used in the study. [file 13071_2020_4001_MOESM1_ESM.pdf]

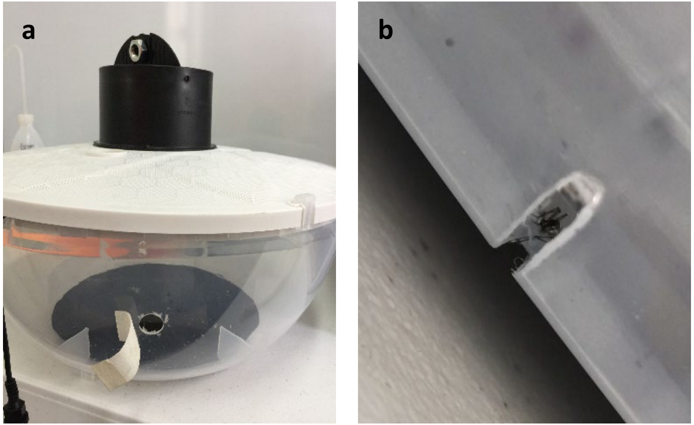

Figure S1: The BG-Bowl used in the study. (**a**) Carbon dioxide is led to the trap via a tube connected to a jerry can where the CO_2 is produced as a by-product of sugar fermentation by yeast. There was no input for CO_2, so an opening was drilled on the side which could be closed off by tape. (**b**) The BG-Bowl has drainage openings at the bottom of the trap. Mosquitoes can escape the trap via the openings, so they were covered with grey duct-tape.
