# Supplementary material for: Optimisation and field validation of odour-baited traps for surveillance of Aedes aegypti adults in Paramaribo, Suriname
Source: Parasit Vectors. 2020 Mar 6;13:121. doi: 10.1186/s13071-020-4001-y (PMC7059684; doi:10.1186/s13071-020-4001-y)
Supplement: Supplementary file 2 — Additional file 2: Table S1. The 4 × 4 Latin Square design used in Field experiment 1. Table S2. The 4 × 4 Latin Square design used in Field experiment 2. [file 13071_2020_4001_MOESM2_ESM.pdf]

Table S1: The 4x4 Latin Square design used in Field experiment 1. The experiment was replicated twice in space and three times in time. Four different treatments were: (A) unbaited trap, (B) CO_2 only (C) MB5 only and (D) CO_2 + MB5.

Day \ Location	1	2	3	4	5	6	7	8
1	A	B	C	D	A	B	C	D
2	D	A	B	C	D	A	B	C
3	C	D	A	B	C	D	A	B
4	B	C	D	A	B	C	D	A
5	A	B	C	D	A	B	C	D
6	D	A	B	C	D	A	B	C
7	C	D	A	B	C	D	A	B
8	B	C	D	A	B	C	D	A
9	A	B	C	D	A	B	C	D
10	D	A	B	C	D	A	B	C
11	C	D	A	B	C	D	A	B
12	B	C	D	A	B	C	D	A

Table S2: The 4 x 4 Latin Square design used in Field experiment 2. The experiment was replicated twice in space and twice in time. All traps were baited with CO_2. Four different treatments were: (A) BG-Sentinel + MB5, (B) BG-Sentinel + BG-Lure, (C) BG-Bowl + MB5, (D) BG-Bowl + BG-Lure.

Day \ Location	1	2	3	4	5	6	7	8
1	A	B	C	D	A	B	C	D
2	D	A	B	C	D	A	B	C
3	C	D	A	B	C	D	A	B
4	B	C	D	A	B	C	D	A
5	A	B	C	D	A	B	C	D
6	D	A	B	C	D	A	B	C
7	C	D	A	B	C	D	A	B
8	B	C	D	A	B	C	D	A
