# Supplementary material for: Optimisation and field validation of odour-baited traps for surveillance of Aedes aegypti adults in Paramaribo, Suriname
Source: Parasit Vectors. 2020 Mar 6;13:121. doi: 10.1186/s13071-020-4001-y (PMC7059684; doi:10.1186/s13071-020-4001-y)
Supplement: Supplementary file 3 — Additional file 3: Table S3. Uncorrected means (%, ± SE) of cage experiment 1. Table S4. Uncorrected means (%, ± SE) of Cage experiment 2. Table S5. Uncorrected means (%, ± SE) of Cage experiment 3. Table S6. Uncorrected means (%, ± SE) of Cage experiment 4. [file 13071_2020_4001_MOESM3_ESM.pdf]

Table S3: Uncorrected means (%, ± SEM) of cage experiment 1. *Aedes aegypti* caught in the inverted BG-Suna trap compared to the BG-Suna trap in normal position.

Treatment	N	Mean (%, ± SEM)
Inverted BG-Suna	8	0.63 ± 0.043
Normal BG-Suna	8	0.37 ± 0.043

Table S4: Uncorrected means (%, ± SEM) of cage experiment 2. *Aedes aegypti* caught in an unbaited inverted BG-Suna trap compared to a BG-Suna trap baited with CO_2, MB5 or MB5 + CO_2.

		Mean (%, ± SEM)	
Treatment	N	Treatment side	Unbaited trap
CO_2 + MB5	4	0.76 ± 0.037	0.24 ± 0.037
CO_2	4	0.70 ± 0.077	0.30 ± 0.077
MB5	4	0.74 ± 0.032	0.26 ± 0.032
Unbaited	4	0.51 ± 0.115	0.49 ± 0.115

Table S5: Uncorrected means (%, ± SEM) of cage experiment 3. *Aedes aegypti* caught in inverted BG-Suna traps baited with CO_2, MB5 or MB5 + CO_2 in a dual-choice experiment.

			Mean (%, ± SEM)	
Treatment	Comparison	N	Treatment side	Comparison side
CO_2 + MB5	CO_2	4	0.70 ± 0.060	0.30 ± 0.060
CO_2	MB5	4	0.66 ± 0.054	0.34 ± 0.054
MB5	CO_2 + MB5	4	0.31 ± 0.099	0.69 ± 0.099

Table S6: Uncorrected means (%, ± SEM) of cage experiment 4. *Aedes aegypti* caught in the CDC-light trap, the MM-X trap, the BG-Sentinel trap and the BG-Suna trap baited with MB5 and CO_2

Treatment	N	Mean (%, ± SEM)
CDC light	8	0.53 ± 0.100
MM-X	8	0.53 ± 0.078
BG-Sentinel	8	0.88 ± 0.053
BG-Suna	8	0.88 ± 0.037
