# Supplementary material for: Optimisation and field validation of odour-baited traps for surveillance of Aedes aegypti adults in Paramaribo, Suriname
Source: Parasit Vectors. 2020 Mar 6;13:121. doi: 10.1186/s13071-020-4001-y (PMC7059684; doi:10.1186/s13071-020-4001-y)
Supplement: Supplementary file 4 — Additional file 4: Table S7. Field experiment 1, mean (± SE) of mosquitoes caught per treatment per day. Table S8. Field experiment 1, P-values of pairwise comparisons (GLM) after LSD correction. Table S9. Field experiment 2, mean (± SE) of mosquitoes caught per treatment per day. Table S10. Field experiment 2, P-values of pairwise comparisons (GLM) after LSD correction. [file 13071_2020_4001_MOESM4_ESM.pdf]

Table S7: Field experiment 1, mean (±SEM) of mosquitoes caught per treatment per day. N = number of data points per treatment.

		Mean (± SEM)			
Treatment	N	*Ae. aegypti* females	*Ae. aegypti* males	*Culex* spp. females	*Culex* spp. males
CO_2 + MB5	20	18.05 ± 3.501	21.31 ± 4.203	49.10 ± 11.240	14.05 ± 4.150
CO_2	23	10.26 ± 2.414	11.04 ± 3.397	19.96 ± 4.109	3.61 ± 0.859
MB5	24	6.13 ± 1.667	11.96 ± 4.047	2.54 ± 0.659	2.88 ± 1.212
Unbaited	24	3.17 ± 0.772	4.83 ± 0.857	2.75 ± 0.748	3.33 ± 1.162

Table S8: Field experiment 1, p-values of pairwise comparisons (GLM) after LSD correction.

		P-values			
Treatments	Comparison	*Ae. aegypti* females	*Ae. aegypti* males	*Culex* spp. females	*Culex* spp. males
CO_2 + MB5	CO_2	0.004	0.005	0.005	0.024
	MB5	< 0.001	0.010	< 0.001	0.011
	Unbaited	< 0.001	< 0.001	< 0.001	0.020
CO_2	MB5	0.029	0.815	< 0.001	0.257
	Unbaited	< 0.001	0.008	< 0.001	0.697
MB5	Unbaited	0.017	0.004	0.847	0.416

Table S9: Field experiment 2, mean (±SEM) of mosquitoes caught per treatment per day. N = number of data points per treatment. All traps were baited with CO_2.

		Mean (±SEM)			
Treatment	N	*Ae. aegypti* females	*Ae. aegypti* males	*Culex* spp. females	*Culex* spp. males
BG-Sentinel + MB5	16	20.50 ± 3.733	30.88 ± 5.875	55.38 ± 10.747	14.06 ± 4.706
BG-Bowl + MB5	16	21.06 ± 5.230	19.88 ± 4.927	36.75 ± 6.337	5.00 ± 1.020
BG-Sentinel + BG-Lure	16	9.44 ± 2.517	12.19 ± 3.089	13.06 ± 4.062	4.00 ± 1.469
BG-Bowl + BG-Lure	16	16.19 ± 4.014	18.31 ± 4.638	18.44 ± 3.858	3.75 ± 0.906

Table S10: Field experiment 2, p-values of pairwise comparisons (GLM) after LSD correction. All traps were baited with CO_2.

		P-values			
Treatments	Comparison	*Ae. aegypti* females	*Ae. aegypti* males	*Culex* spp. females	*Culex* spp. males
BG-Sentinel + MB5	BG-Bowl + MB5	0.362	0.016	0.074	0.025
	BG-Sentinel + BG-Lure	< 0.001	< 0.001	< 0.001	0.001
	BG-Bowl + BG-Lure	0.019	0.009	< 0.001	0.003
BG-Bowl + MB5	BG-Sentinel + BG-Lure	< 0.001	0.008	< 0.001	0.135
	BG-Bowl + BG-Lure	0.143	0.818	0.002	0.315
BG-Sentinel + BG-Lure	BG-Bowl + BG-Lure	0.005	0.015	0.017	0.581
